# Expression Levels of the *Tnni3k* Gene in the Heart Are Highly Associated with Cardiac and Glucose Metabolism-Related Phenotypes and Functional Pathways

**DOI:** 10.3390/ijms241612759

**Published:** 2023-08-14

**Authors:** Qingqing Gu, Buyan-Ochir Orgil, Akhilesh Kumar Bajpai, Yufeng Chen, David G. Ashbrook, Athena Starlard-Davenport, Jeffrey A. Towbin, Djamel Lebeche, Enkhsaikhan Purevjav, Hongzhuan Sheng, Lu Lu

**Affiliations:** 1 Department of Cardiology, Affiliated Hospital of Nantong University, Nantong 226001, China; nt_guqq@126.com (Q.G.); charlie9711@163.com (Y.C.); 2 Department of Genetics, Genomics and Informatics, University of Tennessee Health Science Center, Memphis, TN 38163, USA; abajpai3@uthsc.edu (A.K.B.); dashbroo@uthsc.edu (D.G.A.); astarlar@uthsc.edu (A.S.-D.); 3 The Heart Institute, Department of Pediatrics, University of Tennessee Health Science Center, Memphis, TN 38103, USA; borgil@uthsc.edu (B.-O.O.); jtowbin1@uthsc.edu (J.A.T.); epurevja@uthsc.edu (E.P.); 4 Children’s Foundation Research Institute, Le Bonheur Children’s Hospital, Memphis, TN 38105, USA; 5 Pediatric Cardiology, St. Jude Children’s Research Hospital, Memphis, TN 38105, USA; 6 Department of Physiology, College of Medicine, University of Tennessee Health Science Center, Memphis, TN 38163, USA; dlebeche@uthsc.edu

**Keywords:** *Tnni3k*, BXD family, cardiac physiology, glucose metabolism, systems genetics

## Abstract

Background: Troponin-I interacting kinase encoded by the *TNNI3K* gene is expressed in nuclei and Z-discs of cardiomyocytes. Mutations in *TNNI3K* were identified in patients with cardiac conduction diseases, arrhythmias, and cardiomyopathy. Methods: We performed cardiac gene expression, whole genome sequencing (WGS), and cardiac function analysis in 40 strains of BXD recombinant inbred mice derived from C57BL/6J (B6) and DBA/2J (D2) strains. Expression quantitative trait loci (eQTLs) mapping and gene enrichment analysis was performed, followed by validation of candidate *Tnni3k*-regulatory genes. Results: WGS identified compound splicing and missense T659I *Tnni3k* variants in the D2 parent and some BXD strains (D allele) and these strains had significantly lower *Tnni3k* expression than those carrying wild-type *Tnni3k* (B allele). Expression levels of *Tnni3k* significantly correlated with multiple cardiac (heart rate, wall thickness, PR duration, and T amplitude) and metabolic (glucose levels and insulin resistance) phenotypes in BXDs. A significant *cis*-eQTL on chromosome 3 was identified for the regulation of *Tnni3k* expression. Furthermore, *Tnni3k*-correlated genes were primarily involved in cardiac and glucose metabolism-related functions and pathways. Genes *Nodal*, *Gnas*, *Nfkb1*, *Bmpr2*, *Bmp7*, *Smad7*, *Acvr1b*, *Acvr2b*, *Chrd*, *Tgfb3*, *Irs1*, and *Ppp1cb* were differentially expressed between the B and D alleles. Conclusions: Compound splicing and T659I *Tnni3k* variants reduce cardiac *Tnni3k* expression and *Tnni3k* levels are associated with cardiac and glucose metabolism-related phenotypes.

## 1. Introduction

A highly evolutionary conserved gene among species, cardiac-specific troponin I-interacting kinase *(TNNI3K)* is also known as cardiac ankyrin repeat kinase (CARK). TNNI3K belongs to the mitogen-activated protein kinase kinase kinase (MAPKKK) family of enzymes that play important roles in cardiac function and diseases [1,2]. *TNNI3K* is mainly expressed in the nucleus and sarcomeric Z-discs of fetal and adult cardiac myocytes [1,3]. The 93-kDa TNNI3K is composed of four domains including an ankyrin repeat domain at the N-terminus, followed by a coiled coil domain, a rod kinase domain, and a serine-rich domain at the C-terminus [4]. Importantly, the ankyrin repeat and serine-rich domains have autocatalytic kinase activities and this structural design is similar to that of integrin-linked kinase (ILK), an enzyme involved in cell adhesion and communications between cell and extracellular matrix through integrin-mediated signal transduction [5]. A recent study by Brodehl et al. [6] identified two missense variants (H33N and H77Y) in the ankyrin repeat domain of ILK using whole exome sequencing of arrhythmogenic cardiomyopathy patients. Furthermore, expression of the mutant human ILK in zebrafish demonstrated that H77Y causes cardiac dysfunction and premature death due to heart failure, while H33N mutant cardiomyocytes of adults showed a prolonged action potential. TNNI3K has been reported to bind cardiac troponin I (cTnI), while myosin-binding protein C (MyBPC) and alpha-actin are among the putative binding partners of TNNI3K [1]. Studies have shown that patients with ischemic cardiomyopathy display increased kinase activity of TNNI3K which may promote oxidative stress via impaired mitochondrial function and cardiomyocyte death, whereas inhibition of TNNI3K had protective effects and reduced adverse remodeling in the ischemic heart [7]. A significant positive correlation between *TNNI3K* mRNA levels and electrocardiography (ECG) PR interval duration has been reported in patients with supraventricular tachyarrhythmias [8,9]. Genetic variants L513P, G526D, T539A, and E768K all reduce the kinase activity of TNNI3K, identified in patients with cardiomyopathies and arrhythmias [10,11,12]. Enigmatically, a common single nucleotide polymorphism (SNP), I686T (rs3737564), that is present in up to 4% of human populations, reduced autophosphorylation and kinase activity of TNNI3K in vitro [13], implying potential effects of this kinase in cardiac diseases.

In mice, different strains show distinct levels of *Tnni3k* expression in the heart. For example, DBA/2J (D2) strain carries natural *Tnni3k* variants, T659I (rs30712233) and intronic splicing rs49812611, compared to C57BL/6J (B6) and AKR/J strains [14]. The rs49812611 has been experimentally linked to aberrant TNNI3K protein expression in the D2 strain, which is known to display cardiomyopathy traits including cardiac hypertrophy and fibrosis [14,15]. Importantly, recombinant inbred (RI) mice derived from crosses of B6 and D2 strains, the so-called BXD family of murine genetic reference population (GRP) segregated those cardiomyopathy traits, suggesting a potential relation between cardiomyopathy traits and *Tnni3k* [16]. Knockout B6 mice and transgenic mice with overexpression of mutated kinase-dead TNNI3K demonstrated subtle cardiac hypertrophy compared to mice harboring wild-type *Tnni3k* transgene [3]. Overexpression of kinase-active TNNI3K in murine heart also resulted in pathological cardiac hypertrophy and cardiac dysfunction [3,17], conduction abnormalities [9], ischemia/reperfusion injury [7], and acceleration of cardiomyopathy in the pressure-overload model [14], suggesting that both an increase or a decrease in *Tnni3k* expression could result in cardiomyopathy pathogenesis. Restoration of cardiac *Tnni3k* expression has been associated with cardiomyocyte regeneration and cell cycle activity after myocardial injury [18,19] and promoted cardiomyogenesis and improved cardiac function in mice of the D2 lineage [5]. Nevertheless, genetic and epistatic mechanisms underlying *Tnni3k*’s regulation in the heart remain to be further elucidated.

**Figure 1:**
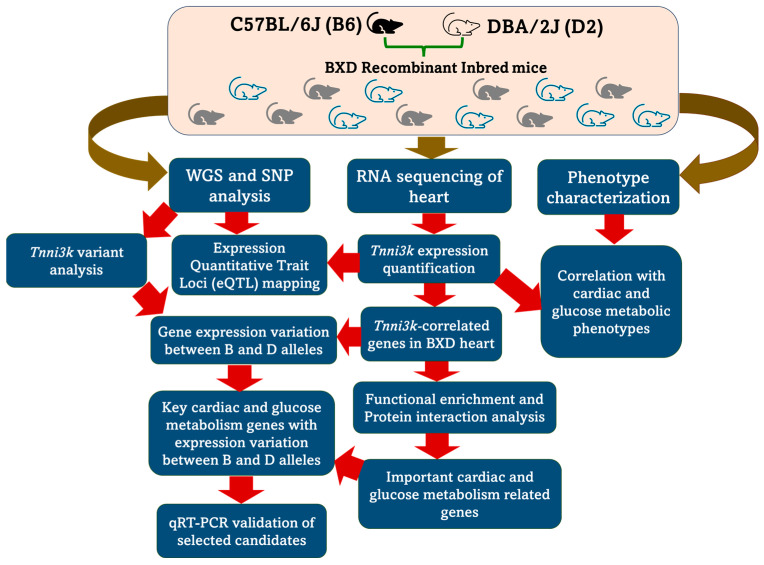
Overall flow of the work. WGS: whole genome sequencing; SNP: single nucleotide polymorphism; qRT-PCR: quantitative real-time reverse transcription polymerase chain reaction.

In this study, we hypothesized that a balanced level of *Tnni3k* expression is essential for proper cardiac function and systems genetics approaches can deliver an understanding of the regulatory mechanisms of the *Tnni3k* expression in the heart through elucidating an association of cardiac function traits with *Tnni3k*. To this end, we utilized parental B6 and D2 mice and 40 BXD strains of murine GRP, a family of RI mice, that shares genotypes from *Tnni3k*-null D2 and control B6 mice for heart gene expression and whole genome sequencing (WGS) analysis and cardiac function assessment using echocardiography and ECG tracings followed by genotype–phenotype association studies. We also applied gene enrichment, functional and pathway analyses to gain insights into the regulatory mechanisms associated with *Tnni3k* function by dissecting its interacting partners and phosphorylation targets. Figure 1 shows the overall flow of the work.

## 2. Results

### 2.1. The Missense Tnni3k T659I Variant Inherent from D2 Parent Segregates among BXDs

The intronic homozygous splicing (rs49812611) and nonsynonymous missense T659I (rs30712233) variants have been identified previously in *Tnni3k*-null D2 parental strain and their effects on *Tnni3k* expression have been validated in vitro [14]. To determine the segregation of these variants in BXD RI strains derived from D2 and B6 mice, we performed whole genome sequencing (WGS) in 40 strains of the BXD family and their parental D2 and B6 strains. A total of 888 variants were identified by WGS in the *Tnni3k* region (154,785,299–155,056,407 bp) in D2 mouse compared to B6, among which, we identified one nonsynonymous (T659I), two synonymous, and one splice variants (chromosome (Chr)3:154,875,191). Of the 40 BXD strains, WGS identified 15 BXD strains to be carrying a compound T659I with the splicing allele from the D2 mouse (named D allele), while other 25 BXD strains had the wild-type T659 *Tnni3k* without the splicing variant (named B allele).

### 2.2. The Strains Carrying the D Allele Have Significantly Reduced Expression of Tnni3k in the Heart

**Figure 2:**
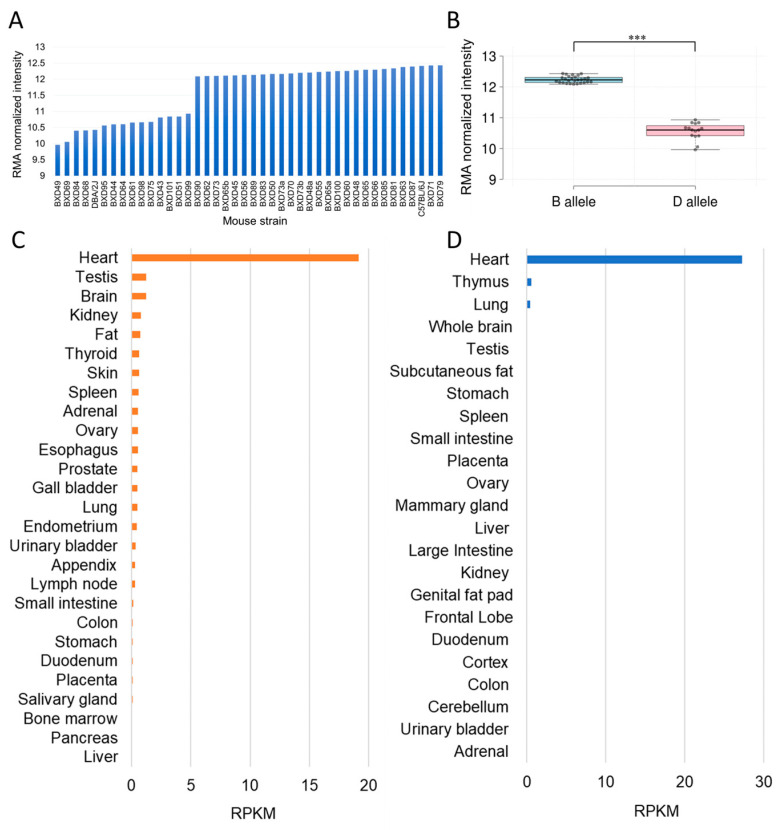
*Tnni3k* expression in mouse and human tissues. (**A**) Expression of *Tnni3k* in the heart tissue of BXD mice and (**B**) between B and D genotypes. The *x*-axis indicates the BXD strains or B and D alleles, and the *y*-axis denotes the RMA (robust multichip average) normalized log2 expression levels. Expression of *Tnni3k* across various adult tissues of (**C**) human and (**D**) mouse based on RNA sequencing data obtained from National Center for Biotechnology Information (NCBI) gene database. The *x*-axis indicates the expression levels in reads per kilobase per million mapped reads (RPKM) and the *y*-axis shows the adult tissue names. *** *p* < 0.0001, indicating a significant difference between B and D alleles.

Next, we explored *Tnni3k* expression in heart tissues from the 40 BXD lines and their parental strains and found *Tnni3k* expression to be highest in BXD79 (12.41), and lowest in BXD49 (9.96) stains, with the mean log2-fold change of *Tnni3k* across 40 BXD strains was 2.47 (5.54 fold). The parental B6 and D2 strains had an expression of 12.41 and 10.42 of normalized robust multichip average (RMA), respectively (Figure 2A), which corresponds to an approximately 4-fold difference between these two strains, similar to the previous report [14]. Furthermore, the expression of *Tnni3k* was found to be significantly different (*p* < 0.0001) between the B and D alleles grouped according to the T659I variant (Figure 2B). The BXD strains carrying the B allele expressed higher levels of *Tnni3k* (median expression: 12.23) than those carrying the D allele (median expression: 10.6). 

We also investigated *Tnni3k* expression based on RNA-seq analysis in multiple tissues in murine and human samples available in the National Center for Biotechnology Information (NCBI) gene database and found it to be specifically expressed in the heart of both species. In the human heart, the *TNNI3K* expression level was 19.2 in reads per kilobase per million mapped reads (RPKM) compared to its mean expression of 0.44 in other adult tissues (Figure 2C). In mouse heart tissue, the expression of *Tnni3k* was 27.2, whereas in other adult tissues, it was either negligible or not detected (Figure 2D), suggesting that *Tnni3k* may play important roles exclusively in heart physiology.

### 2.3. Interval Mapping Methods Identify a cis-Regulating Genetic Locus for Tnni3k

**Figure 3:**
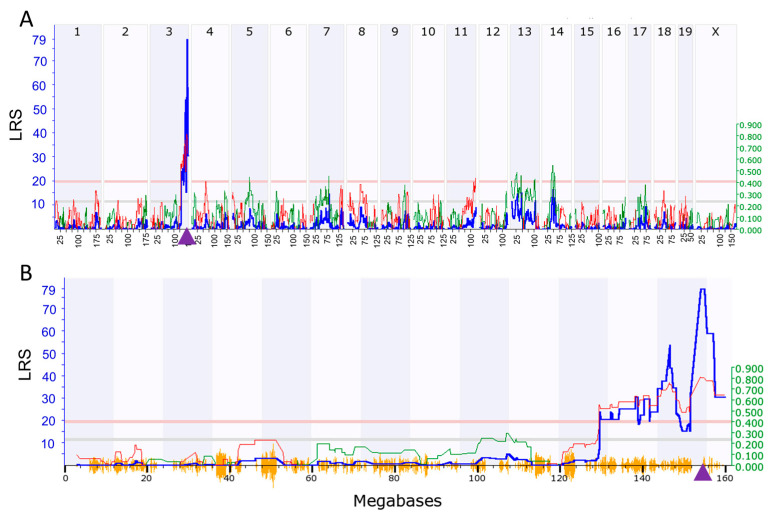
eQTL mapping of *Tnni3k* in the BXD strains. (**A**) Manhattan plots showing the genetic locus on chromosome 3 in murine whole genome that regulates expression of *Tnni3k.* (**B**). Manhattan plots on chromosome (Chr) 3 showing the detailed location of eQTL at 154.78 megabases. Approximately 7300 informative SNP genotype markers across the BXD mouse genome were used for calculating the association with *Tnni3k* mRNA expression variation using likelihood ratio statistics (LRS). The *x*-axis denotes the chromosomal position in megabases on the mouse genome or Chr 3, and *y*-axis indicates the peak LRS score. The pink and grey horizontal lines indicate significant and suggestive LRS, respectively. The purple triangle on the *x*-axis indicates the genomic position of *Tnni3k* (Chr 3: 154.786). LRS is shown by blue lines, and additive effects are shown by red and green lines.

Complex traits are the product of the expression of multiple genes and chromosomal loci that are responsible for variances in expression traits can be discovered by expression quantitative trait loci (eQTL) mapping methods. To identify genetic loci that are responsible for regulating *Tnni3k* expression, we performed eQTL mapping using the interval mapping method on GeneNetwork. Our analysis identified a highly significant eQTL for *Tnni3k* on Chr 3 at 154.78 megabases (Mb) with peak likelihood ratio statistics (LRS) of 79 (Figure 3), the result of which was further confirmed by eQTL mapping using genome-wide efficient mixed-model association (GEMMA) method. As the identified locus is at the chromosomal location of *Tnni3k* (Chr 3: 154.786), it was considered a *cis*-acting eQTL, indicating that the DNA variant in or near *Tnni3k* is responsible for its expression variation.

### 2.4. Tnni3k-Correlated Genes Modulate Pathways Related to Cardiac Physiology and Glucose Metabolism

**Figure 4:**
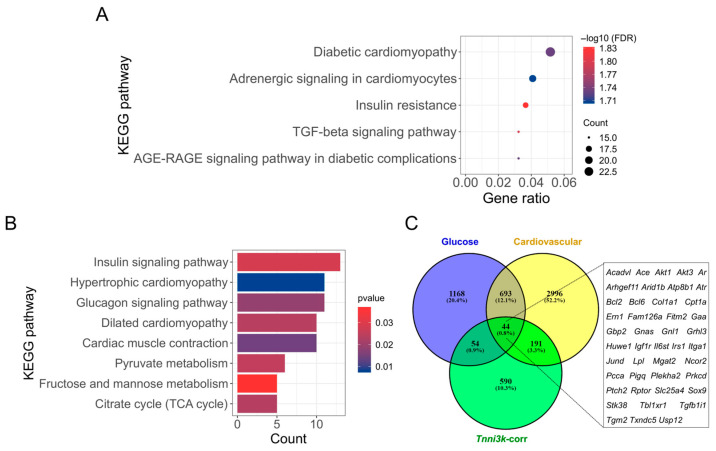
Pathway enrichment analysis of *Tnni3k*-correlated genes. (**A**) Top 5 significantly enriched (FDR *p*-value < 0.02) pathways including diabetic cardiomyopathy, adrenergic signaling in cardiomyocytes, insulin resistance, TGFβ (transforming growth factor beta), and AGE-RAGE signaling in diabetic complications. (**B**) Other significantly enriched (*p*-value < 0.05) cardiac and glucose metabolism-related pathways. The *x*-axis indicates the gene ratio/count, and the *y*-axis represents the KEGG pathways. (**C**) Genetically engineered alleles, transgenes, or QTL variants were retrieved from the Mouse Genome Informatics (MGI) database (http://www.informatics.jax.org/allele, accessed on 24 December 2021) with keywords “glucose metabolism” or “cardiovascular”. The Venn diagram shows the number of overlapping *Tnni3k*-correlated genes involved in “glucose metabolism” and “cardiovascular” related functions.

To understand the role of *Tnni3k* in heart tissue and related diseases, we first identified the genes having a significant correlation with *Tnni3k* expression. The analysis of heart transcriptome data of BXD mice revealed a total of 879 genes to be significantly correlated with *Tnni3k* expression with *p*-value < 0.05, mean expression level > 7, and the literature correlation coefficient > 0.2. We then performed functional and pathway enrichment analysis to define various functions and pathways in which *Tnni3k* correlated genes are involved. The *Tnni3k*-correlated genes were mainly found to be associated with insulin resistance and cardiomyopathy signaling pathways (Figure 4A,B). The top 5 KEGG (Kyoto Encyclopedia of Genes and Genomes) pathways that are significantly enriched by *Tnni3k*-correlated genes (false discovery rate (FDR) *p*-value < 0.02) along with the corresponding gene names are listed in Table 1. Among these, the highest number of correlated genes was found to be enriched in “diabetic cardiomyopathy (DM)” (FDR *p* = 0.0185). Furthermore, “insulin resistance” (FDR *p* = 0.0147), consisting of 17 genes, was the most significant pathway enriched by these correlated genes.

In addition, *Tnni3k*-correlated genes were also enriched (with a *p*-value < 0.05) in cardiomyopathy, cardiac muscle contraction, and glucose metabolism pathways (Figure 4B), including “hypertrophic cardiomyopathy” (*p* = 0.007); “cardiac muscle contraction” (*p* = 0.014); “dilated cardiomyopathy” (*p* = 0.023); “glucagon signaling” (*p* = 0.019); “fructose and mannose metabolism” (*p* = 0.037); “citrate/TCA cycle” (*p* = 0.023); “pyruvate metabolism” (*p* = 0.025); and “insulin signaling” (*p* = 0.029). A list of all significantly enriched KEGG pathways is provided in Supplemental Table S1.

Further, we explored the MGI database to identify the *Tnni3k*-correlated genes that are involved in glucose metabolism and the cardiovascular system, and obtained a total of 1959 and 3924 genes, respectively, with spontaneous, induced, or genetically engineered mutations. Of these, 98 and 235 genes were found to be involved in “glucose metabolism” and “cardiovascular” phenotypes, respectively, and had significant correlations with *Tnni3k* expression. Furthermore, a total of 44 genes of those were associated with both “glucose metabolism” and “cardiovascular” phenotypes (Figure 4C), strengthening the results of our pathway enrichment analysis suggesting that *Tnni3k* modulates cardiovascular phenotypes potentially through glucose metabolic pathways or vice versa.

### 2.5. Nfkb1 Regulates Tnni3k-Correlated Genes Involved in Cardiac-Related and Insulin Resistance Pathways

Previous bioinformatics analysis revealed five potential conserved *cis*-acting transcription factor (TF) binding sites in the core promoter region of *TNNI3K*, of which myocyte enhancer factor-2 (*Mef2*) has been shown as a primary regulator of transcriptional activity of *TNNI3K* [2]. We performed TF enrichment analysis to gain insights into the regulation of genes involved in key pathways and functions related to cardiac physiology. The enrichment analysis of the genes involved in significant KEGG pathways shown in Table 1 found 19 enriched TFs with an adjusted *p*-value < 0.02 (Supplemental Table S2), among which *Nfkb1* was found to be significantly correlated with *Tnni3k* (*p* = 0.035), and targeted eight other genes (*Agt*, *Slc2a1*, *Edn1*, *Akt1*, *Nfkb1*, *Col1a1*, *Bcl2*, and *Bmpr2*) involved in the top 5 KEGG pathways (Table 2). The TRRUST database not only provides the TFs and their targets but also their regulatory relationships. Three of the targets (*Bcl2*, *Nfkb1*, and *Slc2a1*) have been reported to be activated by *Nfkb1*. Interestingly, except *Agt* and *Akt1*, all the other genes targeted by *Nfkb1* shown in Table 2 had significant negative correlations with *Tnni3k*. Aside from *Col1a1*, which was reported to be repressed by *Nfkb1*, the regulatory information for the other target genes was unavailable. Additionally, *Nfkb1* had a mean expression of 10.2 in the heart of BXD mice and had a negative correlation with *Tnni3k* expression (r = −0.32). Thus, the negative correlation of all these genes with *Tnni3k* could be explained owing to the negative correlation of *Nfkb1* with *Tnni3k*, further suggesting a regulatory role for *Nfkb1* in the mechanisms of *Tnni3k* function.

### 2.6. Tnni3k Directly Interacts with Its Correlated Genes Involved in Cardiac and Insulin Resistance-Related Pathways

**Figure 5:**
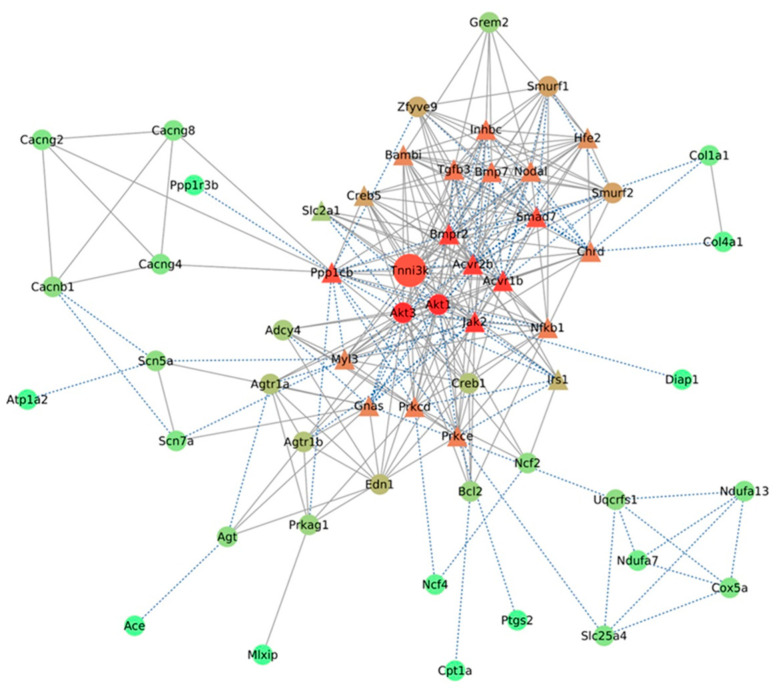
Protein–protein interaction network of *Tnni3k*-correlated genes involved in top key pathways. The PPI network was constructed using *Tnni3k* and its correlated genes involved in the top five pathways identified. Node color (red) intensity represents increasing degree. Triangle nodes represent proteins directly interacting with TNNI3K. The interactions supported by ‘experiments’ evidence in the STRING database are represented with blue dotted lines.

Analysis of protein–protein interaction (PPI) networks helps in understanding the functions of a group of proteins involved in similar pathways or processes. We performed PPI network analysis using *Tnni3k* and its correlated genes involved in the top five pathways (insulin resistance, diabetic cardiomyopathy, AGE-RAGE signaling pathway in diabetic complications, adrenergic signaling in cardiomyocytes, and TGFβ signaling). The PPI network showed 294 interactions among 58 proteins (Figure 5), where TNNI3K directly interacts with 21 proteins in the network, including proteins encoded by 6 genes (*Tgfb3*, *Slc2a1*, *Ppp1cb*, *Prkcd*, *Irs1*, and *Nfkb1*) in “DM”; 4 genes (*Myl3*, *Creb5*, *Ppp1cb*, and *Gnas*) in “adrenergic signaling in cardiomyocytes”; 7 genes (*Creb5*, *Prkce*, *Slc2a1*, *Ppp1cb*, *Prkcd*, *Irs1*, and *Nfkb1*) in “insulin resistance”; 5 genes (*Tgfb3*, *Prkce*, *Prkcd*, *Nfkb1*, and *Jak2*) in “AGE-RAGE signaling pathway in diabetic complications”; and 10 genes (*Bambi*, *Bmpr2*, *Chrd*, *Smad7*, *Acvr2b*, *Bmp7*, *Acvr1b*, *Tgfb3*, *Inhbc*, and *Nodal*) in “TGFβ signaling” pathways.

Notably, NFKB1, the TF which regulates the genes involved in these pathways, was found to be directly interacting with TNNI3K. Additionally, 14 genes encoding the proteins out of those 21 proteins were found to be significantly negatively correlated with *Tnni3k* expression.

### 2.7. Tnni3k-Correlated Genes Involved in Key Pathways Have Significant Expression Differences between B and D Alleles

**Figure 6:**
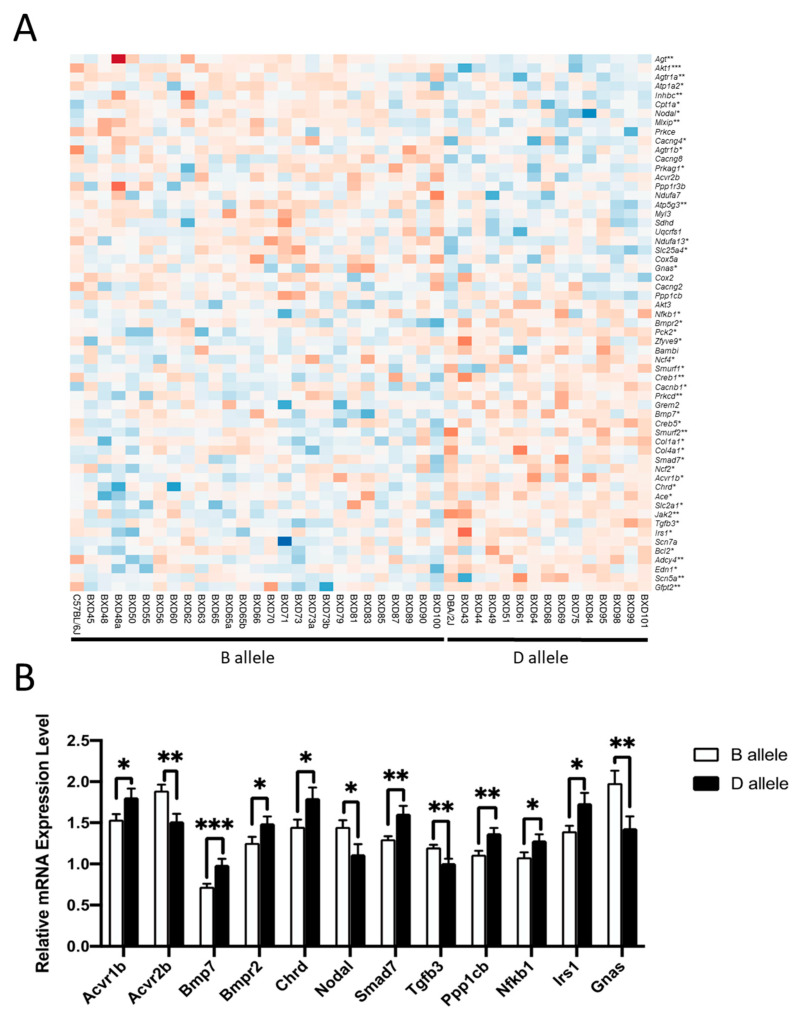
Expression variation of important pathway genes between B and D genotypes. (**A**) The heatmap of genes involved in top five *Tnni3k*-associated pathways constructed using ClustVis tool (https://biit.cs.ut.ee/clustvis/, accessed on 30 December 2021). RMA normalized intensity values across the mice were considered. Each row represents a gene name, and each column indicates a BXD strain grouped according to control B with wild-type T659 *Tnni3k* with no splicing (inherent from parental B6 mouse) or D alleles carrying a compound T659I and splicing (inherent from parental D2 mouse). Genes significantly different between the two genotypes are indicated with an asterisk (* *p* < 0.05, ** *p* < 0.01, *** *p* < 0.001). (**B**) Results of qRT-PCR analysis of myocardial genes that directly interact with *Tinn3k* and have significant expression differences between B and D alleles. BXD strains with wild-type *Tnni3k* represent a B allele (white columns) and those with compound *Tnni3k* variants represent a D allele (black columns). The error bars show standard deviation (SD) values. * *p* < 0.05, ** *p* < 0.01, *** *p* < 0.001.

To explore whether the T659I variant in the *Tnni3k* gene affects the expression of the genes involved in the top five pathways listed in Table 1, we grouped 40 BXD and two parental strains based on their gene expression pattern according to D or B alleles. The results showed that 43 genes of the top five pathways have significant (*p*-value < 0.05) differences between the B and D genotypes, and 13 of those genes (*Agt*, *Akt1*, *Agtr1a*, *Inhbc*, *Mlxip*, *Atp5g3*, *Creb1*, *Prkcd*, *Smurf2*, *Jak2*, *Adcy4*, *Scn5a*, and *Gfpt2*) were significantly different with a *p* < 0.01 (Figure 6A).

We selected 17 significantly differentially expressed genes that directly interact with *Tnni3k* for further validation using quantitative real-time reverse transcription polymerase chain reaction (qRT-PCR) analysis. Of those, 12 genes (*Nodal*, *Gnas*, *Nfkb1*, *Bmpr2*, *Bmp7*, *Smad7*, *Acvr1b*, *Acvr2b*, *Chrd*, *Tgfb3*, *Irs1*, and *Ppp1cb*) were confirmed to have a significant expression difference between the B and D genotypes (Figure 6B).

### 2.8. Levels of Cardiac Tnni3k Expression Significantly Correlate with Cardiac Phenotypes in BXD Mice

**Figure 7:**
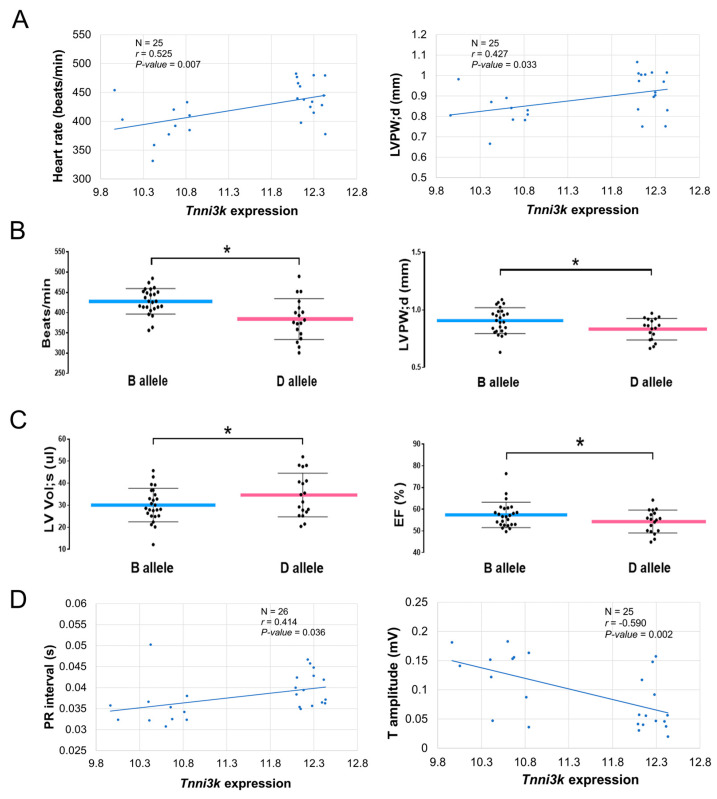
Scatter plots of the correlations between *Tnni3k* expression and echocardiography phenotypes in BXD mice of *Tnni3k* B or D genotypes. (**A**) Correlation between *Tnni3k* expression and echocardiography parameters. *Tnni3k* expression (*x*-axis) is significantly correlated with heart rate (**left panel**, *y*-axis, beats/min) and thickness of left ventricular posterior wall (LVPW) at the end-diastole (d, **right panel**, *y*-axis, mm) in BXD strains (N = 5 mice/strain, 25 strains). The Pearson correlation coefficient (r) was used to determine the relationship. The r- and *p*-values are indicated in each graph. The gene expression level is log2-transformed. (**B**,**C**) Echocardiography parameters between B and D genotypes of BXD mice. *Y*-axis indicates heart rate ((**B**), **left**, beat/min), LVPW thickness ((**B**), **right**, mm), LV volume at the end-systole (s, (**C**), **left**, μL), and ejection fraction (EF, (**C**), **right**, %) in BXD strains of B (25 strains) and D (16 strains) genotypes (N = 5 mice/strain). * *p* < 0.05, indicating a significant difference between B and D alleles. (**D**) Correlation between *Tnni3k* expression and electrocardiography (ECG) tracing parameters. *Tnni3k* expression (*x*-axis) is significantly positively correlated with PR interval (**left panel**, *y*-axis, seconds), whereas significantly negatively correlated with T amplitude (**right panel**, *y*-axis, mV) in BXD strains (N = 5 mice/strain, 25 strains). The r and *p*-values are indicated in each graph. The gene expression level is log2-transformed.

The D2 parental strain of BXD lines carries a compound *Tnni3k* T659I and homozygous splicing which is correlated with *Tnni3k*-null expression (D allele), and the B and D genotypes are segregated among the BXD lines. We, therefore, hypothesized that the variable expression of *Tnni3k* in B and D alleles is correlated with the cardiac phenotypes among BXDs. To test this hypothesis, we performed association analyses between *Tnni3k* expression and cardiac phenotypic parameters which were previously evaluated using echocardiography and ECG tracings in BXDs [16]. Interestingly, we found *Tnni3k* expression to be positively correlated with heart rate (*p* = 0.007, Figure 7A, left) and left ventricular posterior wall thickness at end-diastole (LVPW; d, *p* = 0.033, Figure 7A, right).

Further comparison of echocardiography parameters between B- and D-alleles showed that BXD strains of B-allele had significantly (*p* < 0.05) faster heart rate (Figure 7B, left) and thicker myocardium at the LVPW (Figure 7B, right) compared to that in BXDs of D-allele. In contrast, LV chamber volumes were significantly (*p* < 0.05) lower in the B-allele BXDs (Figure 7C, left). We also found a significant difference in the ejection fraction (EF%) between two alleles with higher systolic function seen in BXDs of the B-allele compared to the D-allele (Figure 7C, right). These results indicated that the BXD strains with higher cardiac *Tnni3k* levels (B allele) have faster heart rates, thicker LV walls, larger LV chambers, and greater systolic (blood pumping) function compared to that in BXD strains of the D genotype carrying compound *Tnni3k* variants.

The association of ECG parameters with *Tnni3k* expression showed a significant positive correlation between *Tnni3k* and duration of PR waves (*p* = 0.036, Figure 7D, left), but negatively correlated with T wave amplitude in BXD strains (*p* = 0.002, Figure 7D, right). Having in mind that BXDs with higher cardiac *Tnni3K* expression have longer PR intervals and lower T amplitude, we compared ECG parameters between different *Tnni3k* genotypes of BXDs; however, no significant differences have been observed between B (25 strains) and D (16 strains) genotypes of BXDs tested. Taken together, results of genotype-phenotype association analysis suggested that *Tnni3k* might be involved in the modulation of heart rate, heart rhythm, myocardial hypertrophy, and systolic function as well as repolarization of ventricular myocardium.

### 2.9. Cardiac Tnni3k Expression Is Significantly Correlated with Glucose Metabolism in BXD Mice

**Figure 8:**
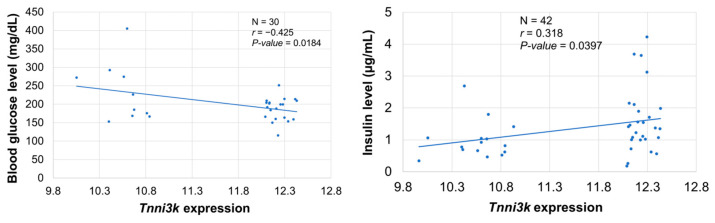
Scatter plots of the correlations between *Tnni3k* expression and glucose metabolism-related phenotypes in BXD mice. (**Left panel**) *Tnni3k* expression (*x*-axis) is significantly negatively correlated with blood glucose level (*y*-axis) in BXD mice (N = 5 mice/strain, 30 strains). (**Right panel**) *Tnni3k* expression (*x*-axis) is significantly positively correlated with insulin response during oral glucose tolerance test (*y*-axis) in BXDs (N = 5 mice/strain, 42 strains). The Pearson correlation coefficient (r) was used to determine the relationship. The r and *p*-values are indicated in each graph. The gene expression level is log2-transformed.

Gene function enrichment analysis of *Tnni3k*-correlated genes identified several significant pathways that were involved in glucose metabolism, suggesting a link between *Tnni3k* in the heart and the regulation of glucose metabolism. Therefore, we correlated glucose metabolism-related phenotypes deposited in our GeneNetwork database with cardiac *Tnni3k* expression in BXD lines. The analysis revealed that cardiac *Tnni3k* expression is negatively correlated with blood glucose level (*p* = 0.018) (Figure 8, left), while positively correlated with insulin response during oral glucose tolerance test in BXD mice (*p* = 0.040) (Figure 8, right). Furthermore, we found that blood glucose and insulin levels were significantly different between mice harboring B and D alleles. While blood glucose levels were higher (*p* = 0.033) in BXD strains harboring D allele (232 mg/dL, n = 10) than in B allele strains (187 mg/dL, n = 20), levels of insulin showed an opposite trend (*p* = 0.043) with 1.60 µg/dL in B (n = 27) and 0.99 µg/dL in D (n = 15) strains.

## 3. Discussion

Although TNNI3K has been investigated for more than two decades since its discovery, reports on its expression, kinase activity, and effects on cardiac phenotypes and function in both humans and mice have been inconsistent and somewhat controversial [20]. Therefore, in this study, we took advantage of the BXD family of murine GRP derived from crosses between B6 and D2 mouse lineages for systems genetics analysis to define the biological roles and regulatory mechanisms of *Tnni3k* by identifying its interacting genes, mediated genetic networks, and molecular signaling pathways through which the kinase function of this gene is modulated. Importantly, a parental D2 strain of the BXD family of RI mice has a natural loss of *Tnni3k* expression compared to the other parental strain, B6. More importantly, a D2 strain has natural cardiomyopathy features relative to a B6 strain that has a normal heart [15] and diverse cardiac morphology and function traits are inherent among BXD RI lines [16]. In this study, our WGS of the BXD family identified that the T659I *Tnni3k* and splicing rs49812611 variants segregated among BXD strains. We found BXD strains carrying a D allele harboring a compound of variants, T659I and splicing rs49812611, have significantly reduced *Tnni3k* expression compared to the BXD strains with B alleles carrying wild-type T659 *Tnni3k* without splicing. The murine T659I corresponds to a very rare human SNP, *TNNI3K* c.1978A > G (T660A, rs1666286197) with a minor allele frequency (MAF) of 0.000004. This allele has not been reported in the Clinvar database and has deleterious effects in silico, predicting its pathogenic nature. Recent studies have identified *TNNI3K* mutations that not only affect its kinase activity but also its expression. For instance, the L513P variant, identified in a young patient with arrhythmogenic right ventricular cardiomyopathy (ARVC), was found to be associated with a decrease in the levels of *TNNI3K’s* mRNA and protein [11]. Based on our current findings, it is conceivable to suggest that the T695I SNP might affect the TNNI3K kinase activity in mice with D alleles, triggering cardiac abnormalities. However, further molecular studies are needed to experimentally validate this prediction and precisely determine whether and how T695I alters kinase activity and expression levels of TNNI3K.

In this study, our eQTL mapping identified a *cis*-acting genetic locus regulating the expression of *Tnni3k*, suggesting that *Tnni3k* has a strong downstream effect on the expression of other genes and cardiac phenotypes. In support of this observation, we found a significant correlation between *Tnni3k* expression and cardiac traits in BXDs including heart rate, PR duration, and T wave amplitude during ECG tracings, and ventricular wall thickness by echocardiography. Furthermore, our study predicted that *Tnni3k* directly interacts with many genes (*Nodal*, *Tgfb3*, *Bmpr2*, *Bmp7*, *Acvr1b*, and *Acvr2b*) that belong to a superfamily of transforming growth factors (TGF) and their receptors. Recruitment of TGFβ activates SMADs (canonical) and noncanonical signaling pathways (TAK, p38) that are involved in cardiac fibrosis and extracellular matrix remodeling in the failing heart [21]. In our study, pathway analysis using *Tnni3k*-correlated genes identified a modulation of not only cardiac but also glucose metabolism-related pathways. Further, we identified *Nfkb1* to be significantly correlated with *Tnni3k-* and *Tnni3k*-correlated genes involved in both, cardiac and insulin resistance-related pathways. NF-κB (nuclear factor kappa light chain enhancer of activated B cells) is a long-known TF to regulate myocardial hypertrophy, cardio-protection, chronic inflammatory response, myocardial apoptosis, post-translational modifications, and its numerous interacting coactivators and corepressors, including histone acetyltransferases (HATs) and histone deacetylases (HDACs) [22]. In contrast, *Tnni3k* was recently shown to be responsible for increased cardiomyocyte S-phase activity after ischemic injury [19], in addition to its involvement in cardiomyocyte regeneration [18]. In line with the significant association between *Tnni3k* expression, *Nfkb1*, and cardiac phenotypes in BXDs, we envision that further molecular studies validating the interaction of TNNI3K with TGFβ and NF-kB on a protein level might demonstrate crucial insights into the function of this kinase.

Diabetes mellitus and heart failure are the leading causes of morbidity and mortality in insulin-resistant diabetic individuals, whereas hyperglycemia and glucotoxicity lead to cardiac injury [23]. Furthermore, chronic hyperglycemia stimulates the production of excessive reactive oxygen species (ROS) by mitochondria, which can induce oxidative damage and apoptosis [24]. Results of our systems genetics analysis revealed a significant association of *Tnni3k* with blood glucose levels (negative correlation) and insulin response to glucose tolerance test (positive correlation) in BXDs. We identified 44 common genes involved in both cardiac and glucose metabolism phenotypes, among which we validated 12 putative direct partners of *Tnni3k* to be significantly differentially expressed between the B and D alleles at the mRNA level. Among the validated genes, *GNAS* encoding the heterotrimeric Gs protein alpha-subunit (Gsα) that increases intracellular cAMP and activates PKA, promoting Ca^2+^ influx and insulin secretion by pancreatic β-cells, has been shown to be involved in both cardiac and metabolic disorders in humans [25]. Maternally inherited *GNAS* mutations cause Albright hereditary osteodystrophy (AHO) and pseudohypoparathyroidism type 1A, whereas paternally inherited mutations cause AHO alone [26]. As revealed by systems biology and population-based studies, a high level of cardiac *GNAS* expression is one of the susceptibility risks for ventricular tachyarrhythmias, sudden cardiac death, and pancreatic β-cell dysfunction [27]. Taken together, we suggest that *GNAS* may be a candidate gene that bridges cardiac and glucose metabolism-related pathways, requiring in-depth validation of its role in cardiometabolic disorders. Therefore, our future studies will be focused on experimental confirmations that cardiac phenotypes are modulated by *Tnni3k* through glucose metabolic pathways.

In summary, we would like to emphasize the uniqueness and importance of using the BXD RI mice population through an integrative omics approach for identifying key genes and underlying molecular mechanisms. The results from the current study may help in discovering better therapeutic molecules for cardiomyopathy by particularly targeting glucose metabolism-related pathways. In addition, a similar approach can be used for exploring genes and mechanisms associated with other pathological/physiological conditions with the help of our murine GRP of the BXD family.

## 4. Materials and Methods

### 4.1. Heart Gene Expression Data in BXD Family

The mice heart gene expression data used in the current study was generated through our collaborative efforts at the University of Tennessee Health Science Center (UTHSC) described in our previous publication [28]. In this study, we used values of cardiac gene expression from forty strains of BXD lines and their B6 and D2 parental strains. The expression data can be accessed through the GeneNetwork website (http://genenetwork.org/, accessed on 17 Decemeber 2021) with the accession number GN485 (EPFL/LISP BXD CD Heart Affy Mouse Gene 2.0 ST Gene Level (Jan14) RMA). Details of tissue collection, RNA isolation, and Affymetrix Mouse Gene 2.0 ST microarray analysis were described previously [28].

### 4.2. Whole Genome Sequencing (WGS) of BXD Family

The anesthetized mice with 5% isoflurane from 40 BXD RI strains and their B6 and D2 parental stains were euthanized by cervical dislocation. Spleen tissue was collected immediately, flash-frozen with liquid nitrogen, and stored at −80 °C for subsequent analysis. The DNA extraction, library preparation, and WGS were carried out by HudsonAlpha (Huntsville, AL, USA). Briefly, genomic DNA was isolated from 50 to 80 mg of spleen tissue using the Qiagen MagAttract Kit (Qiagen, Germantown, MD, USA). The Chromium Gel Bead and Library Kit (v2 HT Kit, revision A; 10X Genomics, Pleasanton, CA, USA) and the Chromium instrument (10X Genomics) were used to prepare libraries for sequencing; barcoded libraries were then sequenced on the Illumina HiSeq X10 system (Illumina, Inc., San Diego, CA, USA).

### 4.3. Variant Bioinformatics Analysis

After WGS was completed, FASTQ files were aligned to the mm10/GRCm38 reference genome using the 10X LongRanger software (v2.1.6) using the “Lariat” alignment approach, and the variant calling was performed using the aligned BAM files through GATK version v3.8-1-0-gf15c1c3ef to generate gVCF files. These gVCFs were then joint-called via Variant Quality Score Recalibration (VQSR) to produce a complete VCF file containing variant calls for all BXDs and their parental stains. The single SNP and indel variant calls from the Sanger Mouse Genomes project were also used as a training set for VQSR. A list of known, “true positive” variants was created for VQSR by identifying variants that were shared across three distinct call sets: variants identified in (1) D2 in the current study, (2) D2 in our previous study [29], and (3) D2 in the Sanger Mouse Genomes Project. This generated a set of 3,972,727 SNPs, 404,349 deletions, and 365,435 insertions that were varied between the D2 and B6 reference sequences. Each of those genetic variations should appear in approximately 50% of the BXD strains. Analyses of all variants identified among BXD strains are publicly available and stored under files in project PRJEB45429 (https://www.ebi.ac.uk/ena/browser/view/PRJEB45429?show=analyses, accessed on 6 August 2023).

### 4.4. Evaluation of Heart Function and Rhythm in BXD Mice

All studies in animals are conducted in accordance with the protocols approved by UTHSC Institutional Animal Care and Use Committee (IACUC). Transthoracic two-dimensional and Doppler echocardiography was performed to evaluate heart function and morphology using a Vevo2100 Micro-imaging System (VisualSonics Inc., Toronto, ON, Canada) as previously described [30]. Echocardiography was performed in 40 strains of BXD mice, B6 and D2 strains at 5–6 months of age (N ≥ 5 mice/strain/sex) and deposited in the GeneNetwork website (https://genenetwork.org/, accessed on 6 August 2023), as we described previously [16], and as displayed in the Supplementary Table S3. Briefly, the chest of the mice was treated with chemical hair remover the day prior. Mice were anesthetized by oxygenated 1–2% isoflurane, and core temperature and heart rate were maintained using a heated platform set at 37 °C. After echocardiography, subcutaneous needle electrodes were placed in the front and rear left legs of the anesthetized mice to obtain consistent ECG recordings, and single-lead ECG tracings were recorded for 5 min at a sampling rate of 200 Hz using BIOPAC (Goleta, CA, USA) and AcqKnowledge 3.9.2 software. All recorded ECGs were imported to the LabChart 7 software for further analysis.

### 4.5. Tnni3k Expression in Multiple Tissues of Mouse and Human

The expression levels of *Tnni3k* based on RNA sequencing data across various human and mouse tissues were obtained from the National Center for Biotechnology Information (NCBI) Gene database (https://www.ncbi.nlm.nih.gov/gene/, accessed on 15 December 2021). The human RNA-seq data corresponded to 95 samples across 27 different tissues [31], whereas the mouse RNA-seq data were generated by the Mouse ENCODE project [32].

### 4.6. Expression Quantitative Trait Locus (eQTL) Mapping

We used the interval mapping method in the webQTL tool on the GeneNetwork website to identify the genomic loci regulating the expression of *Tnni3k*. This method used approximately 7300 informative SNP genotype markers across the BXD mouse genome for calculating the association with *Tnni3k* mRNA expression variation using the likelihood ratio statistics (LRS), and 2000 permutation tests were used to determine the genome-wide suggestive and significance thresholds (suggestive LRS = 11.28, significant LRS = 19.44, and highly significant LRS = 23.97). Finally, the association was evaluated and confirmed with the genome-wide efficient mixed-model association (GEMMA) method [33].

### 4.7. Genetic Correlation Analysis

Genetic correlation analysis was performed on our GeneNetwork portal using the Pearson correlation coefficient to identify gene-phenotype and gene-gene association. For gene–phenotype correlation analysis, we correlated the mRNA level of *Tnni3k* with echocardiography measurements generated in this study and other phenotypes that we generated before and stored in our GeneNetwork database. For gene–gene correlation analysis, we calculated the Pearson correlation between the expression of *Tnni3k* and that of all other genes across the genome and identified sets of genetically *Tnni3k*-correlated genes in the BXD heart. A *p*-value < 0.05 was considered significant. In addition, *Tnni3k*-correlated genes having a mean expression > 7 and a literature correlation coefficient > 0.2 were considered for further analysis. The literature correlation identifies the genes that are described by similar terminologies in titles and abstracts of published articles extracted from MEDLINE/PubMed [34,35].

### 4.8. Pathway Enrichment Analysis

Kyoto Encyclopedia of Genes and Genomes (KEGG) pathway enrichment analysis was performed for the genes that were significantly correlated with *Tnni3k*. The *clusterProfiler* R package with default parameters was used for identifying significantly enriched pathways [36]. The *p*-values were adjusted to account for multiple comparisons using the Benjamini and Hochberg correction [37], and pathways with a *p*-value < 0.05 were considered statistically significant.

### 4.9. Gene Function Analysis

To identify genes having functions in glucose metabolism and the cardiovascular system, we used the Mouse Genome Informatics (MGI: http://www.informatics.jax.org/allele, accessed on 14 December 2021) database [29]. The database was queried with the keywords “glucose metabolism” or “cardiovascular” to obtain mutant or genetically engineered alleles, transgenes, or QTL variants.

### 4.10. Protein–Protein Interaction (PPI) Network Analysis

The protein interactions among the key pathway-associated *Tnni3k*-correlated genes (including *Tnni3k*) were retrieved from the STRING database (www.string-db.org, accessed on 4 December 2021) [38,39]. A minimum score of 0.15 was considered as a threshold for obtaining the interactions. Additionally, only interactions supported by “experiments” and “databases” evidence were considered further. The interacting proteins were analyzed and visualized using Cytoscape [40].

### 4.11. Transcription Factor (TF) Enrichment Analyses

The TF enrichment analysis of the *Tnni3k*-correlated genes involved in key pathways was performed using the TRRUST database (https://www.grnpedia.org/trrust/, accessed on 16 December 2021) [41]. Currently, this manually curated database of human and mouse transcriptional regulatory information contains approximately 6500 TF-target relationships for ~800 mouse TFs. Furthermore, TFs enriched with an FDR-corrected *p* < 0.05 (Benjamini–Hochberg procedure) were considered statistically significant.

### 4.12. Quantitative Real-Time PCR (qRT-PCR)

Five BXD strains carrying the D allele and five BXD strains carrying the B allele as well as D2 and B6 parental strains (N = 3 mice/strain) were used for the validation of *Tnni3k* expression difference between D and B genotypes. Total RNA extracted from mouse hearts using RNeasy Mini Kits (Qiagen) was quantified by NanoDrop. Two micrograms of total RNA were used for cDNA synthesis using High-capacity cDNA Reverse Transcription Kit (Thermo Fisher Scientific, Waltham, MA, USA). Quantitative real-time PCR (qPCR) was performed on Applied Biosystems MiniAMP Plus Thermal Cycler System using PowerUp SYBR Green Master Mix (Thermo Fisher Scientific) as described previously [28]. The primer sequences for mouse transcripts are listed in Supplemental Table S4. The relative expression levels of the genes were calculated using the 2^−ΔΔCt^ method [42]. The fold change for each gene was calculated after normalizing its expression to that of GAPDH, which was used as an internal control.

### 4.13. Statistical Analysis

We grouped the mice based on the presence of the *Tnni3k* T659I variant (D allele) or its absence (B allele) and evaluated the expression of *Tnni3k* and its correlated genes between these genotypes. Statistical significance of the expression variation between the two genotypes was calculated using Student’s *t*-test. Data were analyzed using Data Desk RP8.2.1c (Data Description, Ithaca, NY, USA). A *p*-value of <0.05 was considered statistically significant. GraphPad Prism version 9.0.0 software was used for statistical analysis and graph generation of RT-PCR results.

## 5. Conclusions

In summary, our systems genetics approach demonstrated the importance of *Tnni3k* in both, cardiac and glucose metabolism-related pathways. The importance of *Tnni3k* in these pathways has been further confirmed by the significant correlation of its expression levels with various cardiac phenotypes seen in BXD strains, such as heart rate, thickness of myocardial walls, PR duration, and T amplitude as well as glucose and metabolic traits. NF-kB1 has been found to be a common key TF regulating the expression of *Tnni3k* and *Tnni3k*-correlated genes, while *Gnas* is highly predicted as a common candidate gene for *Tnni3k*-driven cardiometabolic-related pathways.

## Figures and Tables

**Table 1:** Top 5 KEGG pathways significantly enriched by *Tnni3k*-correlated genes with FDR corrected *p*-value < 0.02.

KEGG Pathway	Adjusted *p*-Value	Gene Count	Gene Name
Diabetic cardiomyopathy	0.0185	24	*Atp5g3*, *Slc25a4*, *Ndufa13*, *Uqcrfs1*, *Akt3*, *Sdhd*, *Ncf2*, *Cox5a*, *Ace*, *Ndufa7*, *Ncf4*, *Tgfb3*, *Slc2a1*, *Agtr1b*, *Agt*, *Ppp1cb*, *Agtr1a*, *Gfpt2*, *Prkcd*, *Akt1*, *Col1a1*, *Irs1*, *Nfkb1*, *Cox2*
Adrenergic signaling in cardiomyocytes	0.0198	19	*Akt3*, *Myl3*, *Scn5a*, *Scn7a*, *Atp1a2*, *Creb5*, *Cacng2*, *Agtr1b*, *Agt*, *Ppp1cb*, *Gnas*, *Adcy4*, *Agtr1a*, *Creb1*, *Bcl2*, *Akt1*, *Cacng4*, *Cacnb1*, *Cacng8*
Insulin resistance	0.0147	17	*Akt3*, *Cpt1a*, *Prkag1*, *Mlxip*, *Ppp1r3b*, *Creb5*, *Prkce*, *Slc2a1*, *Agt*, *Ppp1cb*, *Gfpt2*, *Creb1*, *Prkcd*, *Akt1*, *Irs1*, *Pck2*, *Nfkb1*
AGE-RAGE signaling pathway in diabetic complications	0.0185	15	*Akt3*, *Col4a1*, *Tgfb3*, *Edn1*, *Prkce*, *Agtr1b*, *Agt*, *Agtr1a*, *Prkcd*, *Bcl2*, *Akt1*, *Col1a1*, *Nfkb1*, *Diaph1*, *Jak2*
TGFβ signaling pathway	0.0158	15	*Bambi*, *Bmpr2*, *Chrd*, *Smad7*, *Smurf2*, *Acvr2b*, *Smurf1*, *Bmp7*, *Grem2*, *Acvr1b*, *Hjv*, *Tgfb3*, *Inhbc*, *Nodal*, *Zfyve9*

**Table 2:** *Nfkb1*-target genes in the top 5 pathways significantly enriched by *Tnni3k*-correlated genes.

*Nfkb1* Target Genes	KEGG (Kyoto Encyclopedia of Genes and Genomes) Pathway				
	Insulin Resistance	AGE-RAGE Signaling Pathway in Diabetic Complications	Diabetic Cardiomyopathy	Adrenergic Signaling in Cardiomyocytes	TGFβ Signaling Pathway
*Agt*	✓	✓	✓	✓	--
*Slc2a1*	✓	--	✓	--	--
*Edn1*	--	✓	--	--	--
*Akt1*	✓	✓	✓	✓	--
*Nfkb1*	✓	✓	✓	--	--
*Col1a1*	--	✓	✓	--	--
*Bcl2*	--	✓	✓	--	--
*Bmpr2*	--	--	--	--	✓

## Data Availability

All open access links for all available data are highlighted in the manuscript per MDPI Research Data Policies at https://www.mdpi.com/ethics, accessed on 6 August 2023.

## Supplementary Materials

The following supporting information can be downloaded at: https://www.mdpi.com/article/10.3390/ijms241612759/s1.
