# Supplementary material for: Expression Levels of the Tnni3k Gene in the Heart Are Highly Associated with Cardiac and Glucose Metabolism-Related Phenotypes and Functional Pathways
Source: Int J Mol Sci. 2023 Aug 14;24(16):12759. doi: 10.3390/ijms241612759 (PMC10454158; doi:10.3390/ijms241612759)
Supplement: Supplementary file 1 [file ijms-24-12759-s001.zip › Supplementary table s4.pdf]

Supplemental Table S4. The primer sequences of genes that directly connect *Tnni3k* in the PPI network and have significant expression difference between B and D alleles.

Gene	Forward (5'-3')	Reverse (5'-3')
Acvr1b	TCATGATGCGGTCACTGACACC	TCTTTCCCATCACTCGCAAGGC
Acvr2b	GGCTGCGTTTGGAAAGCTC	GCAACAAGTTTTCGTGCTTCA
Bmp7	CGAGACCTTCCAGATCACAGT	CAGCAAGAAGAGGTCCGACT
Bmpr2	GATATGCAGGTTCTTGTGTCCA	TAGCAGTTGACATTGGGTTGAC
Chrd	CAGTGACACAGAAGATTCCT	TTTGAAGGACATCACAGCTC
Nodal	CCTGGAGCGCATTTGGATG	ACTTTTCTGCTCGACTGGACA
Smad7	GAGTCTCCCCCTCCTCCTTA	CAGGCTCCAGAAGAAGTTGG
Tgfb3	ATCAAGAAGAAGAGGGTGGAAGCC	CTCCACTGAGGACACATTGAAACG
Slc2a1	CCATGTATGTGGGAGAGGTGT	TTGCCCATGATGGAGTCTAAG
Ppp1cb	AATATGGAGGTTTTCCACCAGAAG	ATTGATGCTAGCACACTCATGGTT
Prkcd	CTTTGGCAAGGTGCTGCTG	TACCATGGTGGAGAAGCGG
Irs1	TCCCAAACAGAAGGAGGATG	CATTCCGAGGAGAGCTTTTG
Nfkb1	GCTGCCAAAGAAGGACACGACA	GGCAGGCTATTGCTCATCACAG
Creb5	TGTGCCTCCTTGAAACAAGCCATT	ACCAGCATATGCCCAGACTG
Gnas	CTGCCATCATCTTCGTGGTG	GATTTGCCAGCGAGGACTTT
Jak2	TGAAGCAGATAGAGCCAGTCC	TTGGGTGGGTACCAGATCCT
